# Previous abortion and preeclampsia in Kigali, Rwanda: a case-control study

**DOI:** 10.3389/fcvm.2025.1572300

**Published:** 2025-09-09

**Authors:** Anas M. Bakhiet, Nadiah AlHabardi, Ishag Adam

**Affiliations:** ^1^ Faculty of Medicine, University of Medical Science and Technology, Kigali, Rwanda; ^2^ Department of Obstetrics and Gynecology, College of Medicine, Qassim University, Buraidah, Saudi Arabia

**Keywords:** spontaneous abortion, age, preeclampsia, risk factor, pregnancy

## Abstract

**Objective:**

This study was conducted to investigate the association between previous spontaneous abortion and preeclampsia among pregnant women in Rwanda.

**Methods:**

A case (preeclampsia)-control (healthy pregnant women) study with 188 women per group was conducted at Kacyiru Hospital in Kigali, Rwanda. Data were collected using questionnaires, and multivariate binary analysis was performed.

**Results:**

There was no significant difference in age and parity between the case and control groups. In the case group, 29 (15.4%) and 13 (6.9%) women and 16 (8.5%) and 2 (1.1%) women in the control group had a history of one abortion or two or more abortions, respectively. A multivariate binary regression analysis revealed that women with a history of abortion had a higher risk of preeclampsia compared to their peers who had no history of abortion [adjusted odds ratio (AOR) = 2.66, 95% confidence interval (CI): 1.35–5.27]. Women with one past abortion (AOR = 2.33, 95% CI: 1.12–4.86) and those with two or more past abortions (AOR = 8.29, 95% CI: 1.73–39.63) had a higher risk of preeclampsia. Women with a history of preeclampsia who were rural residents or had an increasing body mass index showed a higher risk of preeclampsia.

**Conclusion:**

A history of abortion is associated with preeclampsia. Women with a history of abortion have to receive more frequent care, looking for the development of pre-eclampsia.

## Introduction

Preeclampsia is defined as “the occurrence of hypertension and dysfunction of one or more end organs” (1). It is a worldwide health issue that affects 2%–8% of pregnant women. Preeclampsia is a leading cause of maternal deaths; it is responsible for 10%–15% of direct maternal deaths, the majority of which occur in low-income countries (2). The incidence of preeclampsia has been rising over the last decades, which may be due to factors such as an increase in multiple pregnancies, obesity, and diabetes mellitus, as well as rising maternal age (3). A recent large study in Nigeria (*n* = 71,758) found that 9.5% of women had preeclampsia (4). Preeclampsia has also been reported in 15.7% of women in a specialized hospital in Ethiopia (5) and in 3.2% of pregnant women in the Democratic Republic of the Congo (6).

Despite advances in science that have led to extensive knowledge of the clinical presentation, diagnostic criteria, and management of preeclampsia, the etiology of the condition is not yet fully understood (7), and there is no precise or optimum screening tool or model for identifying women who will develop preeclampsia. The American College of Obstetrics and Gynecology (ACOG) has highlighted several risk factors for preeclampsia, including diabetes mellitus, chronic renal disease, prior chronic hypertension, multiple pregnancies, previous history of preeclampsia, and autoimmune disease (8). Other risk factors associated with preeclampsia include sociodemographic, obstetric, and clinical characteristics such as age, parity, education (4), residence (9), and increased body mass index (BMI) (5, 10). Identifying women who are exposed to these risk factors—and thus vulnerable to developing preeclampsia—is key to providing them with optimum prenatal care or even using preventive measures.

There is ongoing debate about the existence and direction of an association between prior abortion and preeclampsia (4, 11–20). Some studies have shown that prior abortion increased the odds or risk of preeclampsia (4, 11, 13, 15, 21), while other research has reported no effect (16, 17) or even found that prior abortion reduced the odds or risk of preeclampsia (18, 19, 22, 23). Most of these studies were conducted in high-income countries (14, 16, 22, 24). There is a scarcity of data on the history of abortion and preeclampsia in sub-Saharan African countries (13), and none of the studies took place in Rwanda. Since the risk factors for preeclampsia may differ between populations, assessing these risk factors in various populations is necessary. Clinicians, researchers, and health planners must identify the risk factors for preeclampsia, including the history of spontaneous abortion, in numerous settings to identify women who are at higher risk of preeclampsia and provide them with optimum care. Investigating the association between prior abortion and preeclampsia could also help researchers better understand the pathogenesis and immunology of both abortion and preeclampsia.

Preeclampsia is a leading cause of maternal morbidity and mortality (25, 26). However, previous research has reported low usage of World Health Organization (WHO)-recommended practices for the screening and management of preeclampsia and eclampsia by caregivers (antenatal care and labor room consultations) in Rwanda (20). To address the research gap, the present study aims to assess the association between previous spontaneous abortion and preeclampsia among pregnant women in Rwanda.

## Materials and methods

### Study design and setting

A case-control study was conducted at the delivery room of Kacyiru Hospital in Kigali, Rwanda, from February to May 2024. The cases were women with preeclampsia, and an equal number of healthy pregnant women who gave birth in the same hospital were the control group.

### Selection of the cases

The case group consisted of women with preeclampsia, which is defined as “the occurrence of hypertension and dysfunction of one or more end organs” (1). Severe preeclampsia refers to preeclampsia with one of the following features: severe headache, altered cerebral or visual disturbance, hepatic abnormality, renal abnormality, severe hypertension (≥160/110 mmHg), or thrombocytopenia (<100 × 10^9^ platelets/L) (1). Women with systemic disease (e.g., thyroid disease, HIV, diabetes mellitus, renal disease), with multiple gestations in the current pregnancy, or whose fetus had a major congenital malformation were excluded from both the case group and the control group.

After signing an informed consent form, each woman in both the case and control groups was asked to complete a face-to-face questionnaire by two trained medical officers under the supervision of the principal investigator. The questionnaires contained the sociodemographic, clinical, and obstetric history (age, parity, residence, employment status, blood groups, history of preeclampsia, history of miscarriage, and history of preeclampsia). Women with a history of miscarriage were asked about the number of miscarriages. Then, the BMI and sex of the newborn were recorded.

### Sample size

The case group and the control group each contained 188 women (ratio 1:1). The sample size calculation was based on a recent study in Sudan that investigated the association between history of spontaneous abortions and preeclampsia (23). We assumed that 40.0% of the case group would have a history of abortion and 25.0% of the control group would have a history of abortion. The sample size of 188 women per group was established to achieve 80% power and 5% precision. It was assumed that 10% of the women would not respond or would have incomplete data.

## Ethical statement

The current work was conducted according to the Declaration of Helsinki. The present study was approved by the Research Ethics Committee of the Faculty of Medicine, University of Medical Science and Technology, Rwanda. The reference is # 08, 2023. All participants signed written informed consent. The authors followed all measures to ensure participants’ privacy, confidentiality, and safety, such as excluding personal identifiers during data collection.

### Statistical analysis

The statistical analysis was performed using the Statistical Package for the Social Sciences® version 22.0 for Windows (SPSS®; IBM Corp., New York, United States). The proportions of the studied variables were expressed as frequencies (%). The normality of continuous data (age, parity, BMI, and hemoglobin level) was assessed with the Shapiro–Wilk test and was not normally distributed; hence, it was expressed as the median (interquartile range). Multicollinearity (variance inflation factor <4) was evaluated but was not detected among the variables. Univariate binary analysis was performed with preeclampsia as a dependent variable and age, parity, residence, employment status, blood groups, history of preeclampsia, history of miscarriage, BMI, sex of the newborn, and history of preeclampsia as independent variables. Variables with a *p*-value of less than or equal to 0.200 in the univariate analysis were shifted to construct multivariate binary models that considered crude associations between preeclampsia and the variables. Backward elimination (likelihood ratio) was used to adjust the model for covariates. Adjusted odds ratios (AORs) and 95% confidence intervals (CIs) were calculated. A two-sided *p*-value of less than or equal to 0.050 was considered to denote statistical significance.

## Results

There was no significant difference in age and parity between the case group (*n* = 188) and the control group (*n* = 188). BMI and hemoglobin levels were significantly higher in the case group (see Table 1). A significantly higher number of women in the case group were rural residents, were employed, or had a history of preeclampsia. There was no difference in blood groups or sex of the newborn. Forty-two (22.3%) women in the case group and eighteen (9.6%) women in the control group had a history of abortion (*p* = 0.001). Moreover, 29 (15.4%) women and 13 (6.9%) women in the case group and 16 (8.5%) women and 2 (1.1%) women in the control group had a history of one abortion or two or more abortions, respectively (see Table 1). Thus, compared to the control group, a significantly higher number of women in the case group had a history of abortion.

**Table 1:** Univariate analysis of sociodemographic and clinical variables among women with preeclampsia and the control group at Kacyiru hospital in Kigali, Rwanda, 2024.

Variables	Women with preeclampsia (*n* = 188)	Control group (*n* = 188)	Odds (95% confidence interval)	*p*-value
Median (interquartile range)				
Age, years	33.5 (27.0–37.0)	33.0 (28.2–37.0)	0.97 (0.94–1.01)	0.175
Parity	2 (1–3)	2 (1–3)	0.92 (0.81–1.04)	0.180
Body mass index, kg/m^2^	31.2 (27.4–35.6)	27.3 (24.61–29.5)	1.21 (1.15–128)	<0.001
Hemoglobin level, g/dl	12.8 (11.5–13.8)	12.3 (11.4–13.2)	1.16 (1.02–1.32)	0.023
Frequency (proportions)				
Residence				
Urban	153 (81.4)	170 (90.4)	Reference	
Rural	35 (18.6)	18 (9.6)	2.16 (1.17–3.97)	0.013
Employment status				
Housewife	88 (46.8)	109 (58.0)	Reference	
Employed	100 (53.2)	79 (42.0)	1.56 (1.04–2.35)	0.030
History of abortion				
No	146 (77.7)	170 (90.4)	Reference	
Yes	42 (22.3)	18 (9.6)	2.71 (1.49–4.92)	0.001
History of preeclampsia				
No	136 (72.3)	183 (97.3)	Reference	
Yes	52 (27.7)	5 (2.7)	13.9 (5.44–35.97)	<0.001
Number of abortions				
0	146 (77.7)	170 (90.4)	Reference	
One	29 (15.4)	16 (8.5)	2.10 (1.10–4.03)	0.024
≥Two	13 (6.9)	2 (1.1)	7.56 (1.68–34.01)	0.008
Blood group				
Blood group type O	102 (54.3)	111 (59.0)	Reference	
Blood group type other than O	86 (45.7)	77 (41.0)	0.82 (0.54–1. 23)	0.349
Gender of the newborn				
Female	89 (47.3)	86 (45.7)	Reference	0.901
Male	99 (52.7)	102 (54.3)	1.02 (0.68–1.54)	

Shifting the variables with *P* < 0.200 in the univariate analysis to build the multivariate logistic regression analysis (adjusted) and controlling for confounders revealed that women with a history of abortion had a higher risk of preeclampsia compared to their peers who had no history of abortion (AOR = 2.66, 95% CI: 1.35–5.27, *p* = 0.005). When differentiated by the history of abortion, women with a history of one abortion (AOR = 2.33, 95% CI: 1.12–4.86, *p* = 0.023) or two or more abortions (AOR = 8.29, 95% CI: 1.73–39.63, *p* = 0.008) had a higher risk of preeclampsia. Likewise, the risk of preeclampsia was higher among women with a history of preeclampsia (AOR = 1.92, 95% CI: 1.11–3.32, *p* = 0.022), women who were rural residents (AOR = 2.62, 95% CI: 1.28–5.37, *p* = 0.008), and women with an increasing BMI (AOR = 1.21, 95% CI: 1.14–1.28, *p* *<* 0.001; see Table 2). Hemoglobin level was associated with preeclampsia in univariate analysis only (confounder).

**Table 2:** Multivariate analysis of the adjusted odds ratios for the factors associated with preeclampsia at Kacyiru hospital in Kigali, Rwanda, 2024.

Variables		Odds ratio	95.0% confidence interval	*P*
Age, years		0.95	0.91–1.01	0.071
Parity		1.01	0.85–1.19	0.989
Body mass index, kg/m^2^		1.21	1.14–1.28	<0.001
Hemoglobin level, g/dl		1.10	0.96–1.26	0.158
Residence	Urban	Reference		
	Rural	2.62	1.28–5.37	0.008
Employment	Housewife	Reference		
	Employed	1.52	0.95–2.44	0.080
History of preeclampsia	No	Reference		
	Yes	21.60	7.65–61.16	<0.001
History of miscarriage	No	Reference		
	Yes	2.66	1.35–5.27	0.005
Number of miscarriages	0	Reference		
	One	2.33	1.12–4.86	0.023
	≥Two	8.29	1.73–39.63	0.008

## Discussion

The current study indicates that women with a history of abortion had a higher risk of preeclampsia compared to their peers who had no history of abortion (AOR = 2.66). Furthermore, among women with a history of one abortion or two or more abortions, the odds of preeclampsia were increased by 2.33 and 8.29, respectively. These findings are consistent with those of several previous studies (4, 11, 13, 15, 21). For example, in a large study (*n* = 71,758 women) in Nigeria, it was estimated that 6.4% of the women had a hypertensive disorder, and previous abortion was associated with increased odds (OR = 1.22, 95% CI: 1.13–1.30) of hypertensive disorders in pregnancy (4). A history of abortion has similarly been associated with increased odds (AOR = 3.17, 95% CI: 1.31–7.70) of preeclampsia in the Amhara Region in Ethiopia (21). Yemane et al. have observed that previous second-trimester spontaneous abortions were a significant predictor of the progression of gestational hypertension to preeclampsia in the Ayder Comprehensive Specialized Hospital and Mekelle General Hospital in Northern Ethiopia (13). In a large cross-sectional study of 5,170 pregnant women in Tehran, Iran, previous spontaneous abortion was associated with preeclampsia (AOR = 1.28, 95% CI: 1.03–1.59, *p* = 0.025) (11). In a retrospective study that included 492 singleton pregnant women in China, early (first-trimester) spontaneous abortions were associated with an increased risk of preeclampsia (15). In Sweden, research has indicated that one prior miscarriage was not associated with preeclampsia, but two or more miscarriages were associated with an increased risk of preeclampsia (14).

The increased susceptibility to preeclampsia among women who had prior abortions could be explained by or a result of placental dysfunction or early placentation failure, which are features of both miscarriage and preeclampsia (27, 28). The immune intolerance and its triggering effects could result in abortion and may explain the pathogenesis of preeclampsia (29). However, several previous studies have reported that a previous abortion was associated with a reduced risk of preeclampsia (18, 19, 23). A history of spontaneous abortion was associated with a 59.0% reduction in the risk of preeclampsia (AOR = 0.41) in Sudan (23) and a 15.0% decrease in the risk of preeclampsia among primiparous women in China (adjusted relative risk = 0.85) (18). Similarly, Su et al. have observed that a history of induced abortion was associated with a lower risk of preeclampsia among nulliparous women in China (19). In a study conducted in Boston, Massachusetts, in the United States, a history of one induced abortion and a history of three or more induced abortions reduced the risk of preeclampsia by 10% (OR = 0.9) and 30% (OR = 0.7), respectively (22). In findings from Norway, two or more induced abortions decreased the risk of preeclampsia by 64.0% (24). In contrast, one induced abortion has been associated with a borderline reduction of the risk of preeclampsia (95% CI: 0.69–1.02) (24). As mentioned, several studies have reported no association between abortion and preeclampsia (16, 17, 30). The reduced susceptibility to preeclampsia among women with a history of abortion could be explained by the hormonal and immunological changes that occurred in the previous abortion, as they can lead to immune tolerance or adaptation that reduces the risk of preeclampsia (31, 32). The discrepancies in the results of the studies could be due to differences in sociodemographic and genetic characteristics between the study populations. Moreover, some of the studies investigated induced rather than spontaneous abortion, which might have different effects on a woman's immune system.

This study has several limitations. For instance, many factors, such as nutritional and infectious diseases (e.g., HIV), were not assessed in the present study. These diseases have been associated with preeclampsia in previous research (33, 34) and may also be associated with abortion itself. The questionnaire was not validated, and this could question the credibility of the gathered data.

## Conclusion

The present study indicates that a previous abortion increased the risk of preeclampsia. Women with a history of abortion have to receive more frequent care, looking for the development of pre-eclampsia.

## Data Availability

The raw data supporting the conclusions of this article will be made available by the authors, without undue reservation.
